# When Should Premature Ventricular Contractions Be Considered as a Red Flag in Children with Cardiomyopathy?

**DOI:** 10.3390/jcdd8120176

**Published:** 2021-12-10

**Authors:** Marianna Cicenia, Massimo S. Silvetti, Fabrizio Drago

**Affiliations:** Pediatric Cardiology and Arrhythmia/Syncope Complex Unit, Bambino Gesù Children’s Hospital, IRCCS, 00165 Rome, Italy; marianna.cicenia@opbg.net (M.C.); mstefano.silvetti@opbg.net (M.S.S.)

**Keywords:** premature ventricular contractions, children, cardiomyopathy, ventricular dysfunction, arrhythmias

## Abstract

Premature ventricular contractions (PVCs) are common and generally benign in childhood and tend to resolve spontaneously in most cases. When PVCs occur frequently, an arrhythmia-induced cardiomyopathy may be present requiring medical or catheter ablation. PVCs are only rarely the manifestation of a cardiomyopathy. The purpose of this review is to provide some tips and tricks to raise the suspicion of a cardiac disease based on the presence and characteristics of PVCs in children.

## 1. Introduction

Premature ventricular contractions (PVCs) are classically considered common findings among children. Their prevalence varies across reports, case series and the age of children. PVCs are found on electrocardiograms (ECG) and/or 24-h ECG Holter monitoring in approximately 40% of children [1]. Notably, isolated PVCs are detected in about 10–15% of infants with structurally normal hearts and usually disappear in the first three years of life. In contrast, PVCs persist in 20 to 35% of healthy adolescents [2]. Unfortunately, to date, the mechanism relating to the spontaneous resolution of PVCs in childhood remains unclear.

Despite being historically perceived as benign entities, PVCs can sometimes be related to cardiac dysfunction, being the cause or also the consequence. The challenge is to differentiate between benign PVCs, “potentially dangerous” PVCs and malignant PVCs.

## 2. Arrhythmia-Induced Cardiomyopathy

Although PVCs in structurally normal hearts are classically considered benign, arrhythmia-induced cardiomyopathy (CMP) has widely been described in the literature.

The role of PVCs as a cause of left ventricular (LV) dysfunction in the adult population has been extensively recognized, and a PVC burden > 24% has been suggested as the cut-off for predicting the occurrence of PVC-induced CMP. Conversely, this issue is still a matter of intense debate in the pediatric population [3].

In 2010, Kakavand and co-workers reported an average PVC burden associated with LV dysfunction in children of 36% with the recovery of the cardiac function in all patients after a successful treatment or spontaneous resolution of PVCs [4]. More recently, Bartels demonstrated that a PVC burden > 30% was significantly associated with the development of LV dysfunction in children [5].

Differently from these results, Guerrier et al. did not report any significant relationships between the PVC burden and LV systolic function in a cohort of patients <21 years old, nor with the PVC morphology, coupling interval or complex PVCs (couplets, triplets or short runs of nonsustained ventricular tachycardia (nsVT)) [6]. These findings were confirmed by Abadir et al., who demonstrated, however, a strong correlation between the PVC coupling interval and LV dysfunction with a cut-off value of 365 ms [7]. This further supported the data of Sun and colleagues who found a correlation between frequent PVCs (>10/min) and a short coupling interval (RR’ ≤ 0.6), both causing a marked reduction of the ejection fraction and cardiac indices [8].

Although a strong correlation with the PVC burden or other factors has not been demonstrated as the underlying mechanism of PVC-induced CMP, possible explanations could be (i) transient changes in intracellular calcium, (ii) the maintenance of an ionic flux during the abnormal contraction, and (iii) LV dyssynchrony, which may lead to LV dilatation and dysfunction, reduced cardiac output and coronary perfusion [4,9,10].

Based on these considerations, Drago et al. recommend the treatment of idiopathic PVCs with antiarrhythmic drugs or a catheter ablation, only in the presence of a depressed LV function or to reduce symptoms [11].

## 3. Malignant PVCs

Despite being most often considered benign, PVCs in children may also be the epiphenomenon of a CMP, which can also precede the onset of CMP, or the manifestation of a channelopathy.

The risk of life-threatening arrhythmias is a well-established concept in CMPs such as hypertrophic cardiomyopathy (HCM) and arrhythmogenic cardiomyopathy (ACM) [12,13].

As for idiopathic dilated CMP, isolated or complex PVCs develop quite late, and therefore, to date, the only parameter considered for arrhythmic risk and primary prevention is a marked reduction in the ejection fraction. Indeed, in current guidelines the absolute benefit of the implantable cardioverter-defibrillator (ICD) in nonischemic dilated cardiomyopathy (non-IDCM) is considered lower than in IDCM as the class of recommendation for ICD implantation has changed from class I to class IIa for symptomatic patients with non-IDCM and an ejection fraction ≤ 35% despite at least three months of optimal medical therapy [14,15].

Very recently, a greater arrhythmogenic risk has been observed in patients with CMP such as arrhythmogenic ventricular CMP (right and left dominant) as well as in end-stage HCM and LV noncompaction. CMP with an arrhythmic phenotype is currently classified as “arrhythmogenic” cardiomyopathy (ACM). This arrhythmic phenotype can occur even in the absence of overt heart failure, and the prognosis is not related to the severity of right ventricular (RV)/LV dysfunction and dilatation [16].

The common hallmark of these forms of ACM is a large amount of fibrosis with a subsequent propensity for cardiac arrhythmias, even in the initial stages. The left dominant forms may present with a “hypokinetic, nondilated phenotype”, which means that a mild or more than mild hypokinesia may be present even in the absence of dilatation. This differs from the classic idiopathic DCM where dilatation and marked impaired systolic function are the key features of the disease and where the absence of extended fibrosis only confers a significant arrhythmic risk during the advanced stages of heart failure [17].

ACM, aside from arrhythmogenic right ventricular dysplasia, can also be due to ACM with left dominance and desmosomal mutations as well as to CMP with mutations of non-desmosomal genes such as TMEM 43 and TGFβ, which interact with the function of desmosomes (i.e., LMN A/C, FLNC, PLN), conferring an elevated arrhythmic risk. Furthermore, secondary CMPs characterized by fibrosis and arrhythmic risk (e.g., chronic myocarditis, Chagas’ disease, cardiac sarcoidosis and amyloidosis, and histiocytic, mitochondrial or metabolic forms of CMP) can be classified in the same way.

Notably, channelopathies can also cause ACM. Pathologic variants in genes encoding for ion channel proteins and causing inherited arrhythmic disorders (e.g., long QT syndrome, short QT syndrome, Brugada syndrome, catecholaminergic polymorphic ventricular tachycardia, etc.) have been described as having a possible role in the development of ACM [18].

In all these cases, the family medical history, symptoms and PVC characteristics are of paramount importance. For this reason, a family history of cardiomyopathy, channelopathy or sudden cardiac death should raise concerns when evaluating children with PVCs.

Symptoms such as palpitations, chest discomfort, fatigue and syncope, especially during effort, should be carefully evaluated in the diagnostic work-up, as they often suggest an underlying cardiac disease. Moreover, if the PVC burden and complexity tend to progress and increase during follow-up, this should raise the suspicion of a cardiac disease [19].

## 4. Evaluation of Pediatric Patients with PVCs: Tips and Tricks

Our approach to the evaluation of pediatric patients with PVCs includes: ECG, 24-h ECG Holter monitoring, exercise testing, echocardiogram, and eventually advanced imaging and genetic testing.

The ECG is useful to rule out any pathological findings that may be associated with PVCs due to a CMP, including a T-wave inversion in the precordial leads, low voltages in the limb leads, the epsilon wave, a prolonged terminal activation of the QRS, signs of ventricular hypertrophy or remodeling.

24-h ECG Holter monitoring is useful for quantifying the PVCs’ burden, for evaluating complex PVCs, nsVT or sustained ventricular tachycardias (sVT) as well as for assessing the prevalence of PVCs during daytime or nighttime, or an eventual polymorphism.

Exercise testing is crucial for evaluating the response to physical activity, i.e., whether PVCs are suppressed, increased on effort, or persist or appear during the recovery phase. This latter finding is still a matter of debate when trying to classify PVCs as benign or not. In fact, the response to exercise could help in differentiating between malignant and benign PVCs. Classically, PVCs that are suppressed or decrease with exercise are considered benign while PVCs occurring or increasing at a high workload have been considered a warning sign of heart disease. As is well known, the appearance of bidirectional polymorphic ventricular contractions/VT during exercise should raise the suspicion of catecholaminergic polymorphic ventricular tachycardia [20,21]. However, in specific situations the role of the exercise test is questionable. In this regard, Sequeira reported that, in young patients with suspected ACM, the absence or suppression of PVCs during exercise should not necessarily be considered a benign sign and that the role of exercise testing in this setting remains to be clearly established [22]. Notwithstanding this, some authors suggest further investigations in the presence of an uncommon PVC pattern or of rare, repetitive, polymorphic, short-coupled and exercise-induced PVCs [23].

During exercise testing, it is also important to rule out variations of the ST-T segment as an ischemic sign due to coronary disease (coronary anomaly or intramyocardial bridge) or, in the case of HCM, due to sub-endocardial ischemia.

Regarding the site of origin of PVCs, the outflow tract is classically considered the most common site in idiopathic/benign PVCs (typical left bundle branch block (LBBB) morphology with inferior axis). Differentiating PVCs arising from the right ventricular outflow tract (RVOT) from those of the left ventricular outflow tract (LVOT) may be challenging. An early transition in the precordial leads suggests an LVOT origin.

**Figure 1:**
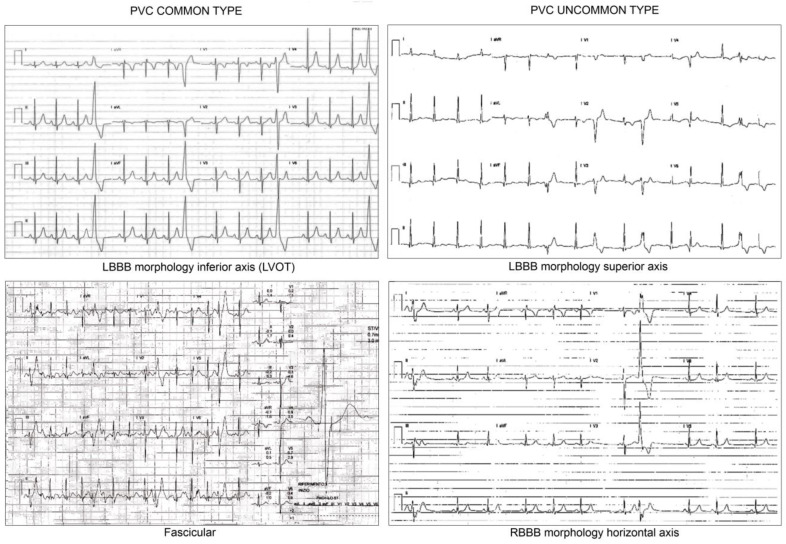
ECG pattern morphologies. Legend: LBBB: left bundle branch block; LVOT: left ventricular outflow tract; RBBB: right bundle branch block.

PVCs with a wide QRS (>130 ms) and LBBB morphology and with a horizontal, intermediate or indeterminate axis suggest the right ventricular free wall as the site of origin. In this case, a more thoroughly investigation is needed as PVCs may be associated with structural heart muscle disease. Additionally, PVCs > 500/24 h with an LBBB non-RVOT morphology can be considered a major criteria for ACM diagnosis according to the new diagnostic criteria [24] (Figure 1).

In children, a fascicular origin of PVCs (right bundle branch block (RBBB), narrow QRS, and superior axis when originating from the posterior fascicle or inferior axis when originating from the anterior fascicle) should be considered a reassuring feature. Conversely, PVCs with a wide QRS and atypical RBBB morphology and with a horizontal or indeterminate axis should trigger a thorough evaluation as they may be a sign of impaired LV function (fibrosis, cardiac masses, mitral valve prolapse).

Notably, benign monomorphic VTs have been shown in patients with PVCs originating from the RVOT, fascicles or papillary muscles [19,25,26], though papillary muscle ventricular arrhythmias may induce PVC-mediated CMP [27].

As fascicular PVCs carry a benign prognosis and tend to disappear during childhood, some authors recommend the same follow-up approach as for PVCs of LBBB morphology, which conversely tend to remain unchanged with age, though CMP may developing over time in some cases despite a benign diagnosis at initial evaluation [28,29].

An “atypical” PVC morphology, less numerous PVCs, and repetitive, polymorphic and incessant VT can also be the manifestation of hamartomas, CMPs, cardiac tumors, myocarditis, and mitral valve prolapse [2,23] (Table 1).

Two-dimensional transthoracic echocardiography is the first-line imaging modality for ruling out cardiac dysfunction, cardiac hypertrophy or other pathological conditions such as intracardiac masses (e.g., fibromas, rhabdomyomas, hamartomas, Purkinje cell tumors, etc.). Unfortunately, in several CMPs, including ACM, it is of very limited usefulness except for the initial stages.

Two-dimensional speckle tracking strain analysis may be useful for detecting a subtle reduction of the ejection fraction or segmental wall motion abnormalities, but further studies are warranted to validate these parameters as diagnostic tools [30,31].

If the suspicion of a malignant arrhythmia remains high, second-line tests are essential to rule out the presence of structural heart disease/CMP/myocarditis/cardiac masses.

Contrast-enhanced cardiac magnetic resonance (CMR) allows for the assessment of the RV/LV dimensions as well as global and regional systolic function, specifically looking at abnormal wall thinning, RVOT dilation, RV/LV enlargement, biventricular global dysfunction, regional wall motion abnormalities, and the presence of late gadolinium enhancement [32,33]. The importance of CMR is one of the crucial points of the updated “Padua criteria” for ACM [24], but its role is unquestionable in all other CMPs.

**Figure 2:**
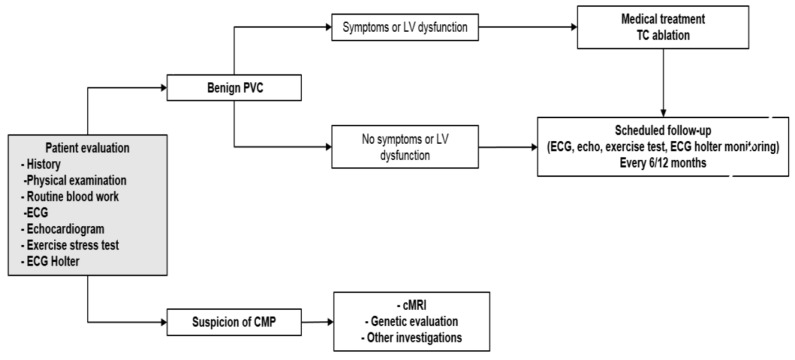
Diagnostic workflow of patients with premature ventricular contractions. Legend: CMP: Cardiomyopathy; cMRI: Cardiac magnetic resonance imaging; ECG: electrocardiogram; LV: left ventricular; PVC: Premature ventricular contractions; TC: transcatheter.

Finally, genetic testing is indicated in selected cases to confirm or refine the diagnosis and risk stratification of CMPs, especially if they are genetically determined, and to eventually enable an appropriate family screening [34] (Figure 2).

## 5. Treatment Options

As previously reported, benign PVCs should only be treated in the case of arrhythmia-induced CMP or to reduce symptoms [11]. Beta-blockers and class IC antiarrhythmic drugs are the preferred agents, whereas class III antiarrhythmic drugs may be used in selected cases. Catheter ablation may be considered in patients who are refractory to medical therapy or those with a progressive impairment of the LV function [11,25].

If PVCs are associated with channelopathies or CMPs, the treatment depends on the underlying disease mechanism and is mainly aimed at preventing sudden cardiac death. According to current guidelines, pharmacological treatment is the first-line approach, and catheter ablation may be indicated in selected cases with CMPs, taking into account the progressive nature of the disease. Finally, ICD implantation is often considered the only effective treatment for the prevention of sudden cardiac death in this particular patient population [35].

## 6. Conclusions

In children, PVCs are often a benign phenomenon and rarely the manifestation of a cardiac disease.

Red flags that should prompt further investigation include the following:

− Positive family history
− Very frequent PVCs
− Polymorphic PVCs
− Uncommon PVC pattern (nonfascicular/non-outflow PVC)
− PVCs not suppressed by effort

## Figures and Tables

**Table 1:** Characteristics of benign and malignant premature ventricular contractions (PVCs).

Benign PVCS	Malignant PVCS
Negative familial history	Positive familial history
Mild/No symptoms	No/Mild/Severe symptoms
Monomorphic	Monomorphic or polymorphic
Common ECG pattern	Uncommon ECG pattern
Suppressed by exercise	Induced, suppressed or not by exercise

## Data Availability

The data presented in this study are available on request from the corresponding author. The data are not publicly available due to Institutional and Research policies.

## References

[1] Massin M.M., Bourguignont A., Gérard P. (2005). Study of Cardiac Rate and Rhythm Patterns in Ambulatory and Hospitalized Children. Cardiology.

[2] Alexander M.E., Berul C. (2000). Ventricular Arrhythmias: When to Worry. Pediatr. Cardiol..

[3] Baman T.S., Lange D.C., Ilg K.J., Gupta S.K., Liu T.-Y., Alguire C., Armstrong W., Good E., Chugh A., Jongnarangsin K. (2010). Relationship between burden of premature ventricular complexes and left ventricular function. Hear. Rhythm..

[4] Kakavand B., Ballard H.O., Disessa T.G. (2010). Frequent Ventricular Premature Beats in Children with a Structurally Normal Heart: A Cause for Reversible Left Ventricular Dysfunction?. Pediatr. Cardiol..

[5] Bertels R.A., Harteveld L.M., Filippini L.H., Clur S.A., Blom N.A. (2017). Left ventricular dysfunction is associated with frequent premature ventricular complexes and asymptomatic ventricular tachycardia in children. EP Eur..

[6] Guerrier K., Anderson J.B., Czosek R.J., Mays W.A., Statile C., Knilans T.K., Spar D.S. (2015). Usefulness of Ventricular Premature Complexes in Asymptomatic Patients ≤21 Years as Predictors of Poor Left Ventricular Function. Am. J. Cardiol..

[7] Abadir S., Blanchet C., Fournier A., Mawad W., Shohoudi A., Dahdah N., Khairy P. (2016). Characteristics of premature ventricular contractions in healthy children and their impact on left ventricular function. Hear. Rhythm..

[8] Sun Y., Blom N.A., Yu Y., Ma P., Wang Y., Han X., Swenne C.A., van der Wall E.E. (2003). The influence of premature ventricular contractions on left ventricular function in asymptomatic children without structural heart disease: An echocardiographic evaluation. Int. J. Cardiovasc. Imaging.

[9] Niwano S., Wakisaka Y., Niwano H., Fukaya H., Kurokawa S., Kiryu M., Hatakeyama Y., Izumi T. (2009). Prognostic significance of frequent premature ventricular contractions originating from the ventricular outflow tract in patients with normal left ventricular function. Heart.

[10] Wilber D.J. (2009). Ventricular ectopic beats: Not so benign. Heart.

[11] Fabrizio D., Loira L., Gabriele B., Berardo S., Giulio P. (2017). Premature ventricular complexes in children with structurally normal hearts: Clinical review and recommendations for diagnosis and treatment. Minerva Pediatr..

[12] Norrish G., Ding T., Field E., Ziolkowska L., Olivotto I., Limongelli G., Anastasakis A., Weintraub R., Biagini E., Ragni L. (2019). Development of a Novel Risk Prediction Model for Sudden Cardiac Death in Childhood Hypertrophic Cardiomyopathy (HCM Risk-Kids). JAMA Cardiol..

[13] Calkins H., Corrado D., Marcus F. (2017). Risk Stratification in Arrhythmogenic Right Ventricular Cardiomyopathy. Circulation.

[14] Ponikowski P., Voors A.A., Anker S.D., Bueno H., Cleland J.G.F., Coats A.J.S., Falk V., Gonzalez-Juanatey J.R., Harjola V.-P., Jankowska E.A. (2016). 2016 ESC Guidelines for the diagnosis and treatment of acute and chronic heart failure: The Task Force for the diagnosis and treatment of acute and chronic heart failure of the European Society of Cardiology (ESC)Developed with the special contribution of the Heart Failure Association (HFA) of the ESC. Eur. J. Heart Fail..

[15] McDonagh T.A., Metra M., Adamo M., Gardner R.S., Baumbach A., Böhm M., Burri H., Butler J., Čelutkienė J., Chioncel O. (2021). 2021 ESC Guidelines for the diagnosis and treatment of acute and chronic heart failure. Eur. Heart J..

[16] Spezzacatene A., Sinagra G., Merlo M., Barbati G., Graw S.L., Brun F., Slavov D., di Lenarda A., Salcedo E.E., Towbin J.A. (2015). Arrhythmogenic Phenotype in Dilated Cardiomyopathy: Natural History and Predictors of Life-Threatening Arrhythmias. J. Am. Hear. Assoc..

[17] (2021). Differential diagnosis of arrhythmogenic cardiomyopathy: Phenocopies versus disease variants. Minerva Med..

[18] Towbin J.A., McKenna W.J., Abrams D., Ackerman M.J., Calkins H., Darrieux F., Daubert J.P., de Chillou C., de Pasquale E.C., Desai M.Y. (2019). 2019 HRS expert consensus statement on evaluation, risk stratification, and management of arrhythmogenic cardiomyopathy. Hear. Rhythm..

[19] Cohen M. (2019). Frequent premature ventricular beats in healthy children: When to ignore and when to treat?. Curr. Opin. Cardiol..

[20] Roston T.M., Yuchi Z., Kannankeril P.J., Hathaway J., Vinocur J.M., Etheridge S.P., Potts J., Maginot K.R., Salerno J.C., Cohen M. (2018). The clinical and genetic spectrum of catecholaminergic polymorphic ventricular tachycardia: Findings from an international multicentre registry. EP Eur..

[21] Pflaumer A., Wilde A.A., Charafeddine F., Davis A.M. (2020). 50 Years of Catecholaminergic Polymorphic Ventricular Tachycardia (CPVT)—Time to Explore the Dark Side of the Moon. Hear. Lung Circ..

[22] Sequeira I.B., Kirsh J.A., Hamilton R.M., Russell J.L., Gross G.J. (2009). Utility of Exercise Testing in Children and Teenagers with Arrhythmogenic Right Ventricular Cardiomyopathy. Am. J. Cardiol..

[23] Corrado D., Drezner J.A., D’Ascenzi F., Zorzi A. (2019). How to evaluate premature ventricular beats in the athlete: Critical review and proposal of a diagnostic algorithm. Br. J. Sports Med..

[24] Corrado D., Perazzolo Marra M., Zorzi A., Beffagna G., Cipriani A., Lazzari M., Migliore F., Pilichou K., Rampazzo A., Rigato I. (2020). Diagnosis of arrhythmogenic cardiomyopathy: The Padua criteria. Int. J. Cardiol..

[25] Higuchi K., Bhargava M. (2021). Management of premature ventricular complexes. Heart.

[26] Anderson R.D., Kumar S., Parameswaran R., Wong G., Voskoboinik A., Sugumar H., Watts T., Sparks P.B., Morton J.B., McLellan A. (2019). Differentiating Right- and Left-Sided Outflow Tract Ventricular Arrhythmias: Classical ECG signatures and prediction algorithms. Circ. Arrhythmia Electrophysiol..

[27] Busch S., Eckardt L., Sommer P., Meyer C., Bonnemeier H., Thomas D., Neuberger H.-R., Tilz R.R., Steven D., von Bary C. (2019). Ventrikuläre Extrasystolen und Tachykardien bei strukturell normalem Herz. Herzschrittmachertherapie Elektrophysiol..

[28] Beaufort-Krol G.C., Dijkstra S.S., Bink-Boelkens M.T. (2008). Natural history of ventricular premature contractions in children with a structurally normal heart: Does origin matter?. EP Eur..

[29] Jiang J., He Y., Qiu H., Zhang Y., Chu M., Li Y., Chen Q. (2017). Analysis of Morphological Characteristics and Origins of Idiopathic Premature Ventricular Contractions Under a 12-Lead Electrocardiogram in Children with Structurally Normal Hearts. Int. Hear. J..

[30] Pinamonti B., Abate E., de Luca A., Finocchiaro G., Korcova R., Sinagra G., Merlo M. (2019). Role of Cardiac Imaging: Echocardiography. Idiopathic Dilated Cardiomyopathy.

[31] Pieles G.E., Grosse-Wortmann L., Hader M., Fatah M., Chungsomprasong P., Slorach C., Hui W., Fan C.-P.S., Manlhiot C., Mertens L. (2019). Association of Echocardiographic Parameters of Right Ventricular Remodeling and Myocardial Performance With Modified Task Force Criteria in Adolescents With Arrhythmogenic Right Ventricular Cardiomyopathy. Circ. Cardiovasc. Imaging.

[32] Tandri H., Castillo E., Ferrari V.A., Nasir K., Dalal D., Bomma C., Calkins H., Bluemke D.A. (2006). Magnetic resonance imaging of arrhythmogenic right ventricular dysplasia: Sensitivity, specificity, and observer variability of fat detection versus functional analysis of the right ventricle. J. Am. Coll. Cardiol..

[33] Geva T. (2015). Imaging criteria for arrhythmogenic right ventricular cardiomyopathy: An incomplete journey. J. Am. Coll. Cardiol..

[34] Hershberger R.E., Givertz M.M., Ho C.Y., Judge D.P., Kantor P.F., McBride K.L., Morales A., Taylor M.R.G., Vatta M., Ware S.M. (2018). Genetic evaluation of cardiomyopathy: A clinical practice resource of the American College of Medical Genetics and Genomics (ACMG). Genet. Med..

[35] Priori S.G., Blomström-Lundqvist C., Mazzanti A., Blom N., Borggrefe M., Camm J., Elliott P., Fitzsimons D., Hatala R., Hindricks G. (2015). 2015 ESC Guidelines for the management of patients with ventricular arrhythmias and the prevention of sudden cardiac death of the European Society of Cardiology (ESC). Endorsed by: Association for European Paediatric and Congenital Cardiology (AEPC). EP Eur..

